# Characteristics and risk factor analysis of 410 cases of tracheobronchial tuberculosis

**DOI:** 10.3892/etm.2014.1804

**Published:** 2014-06-24

**Authors:** XINMEI GUO, CHUNYAN WANG, XIAOPING WANG, JUAN MA, LI XV, TINGTING LUAN, CHANGWEI KOU

**Affiliations:** Tuberculosis Division, Shandong Chest Hospital, Jinan, Shandong 250013, P.R. China

**Keywords:** tuberculosis, trachea, bronchus, bronchoscopy, chest computed tomography, etiology, prognosis

## Abstract

The present study analyzed the characteristics and risk factors associated with tracheobronchial tuberculosis (TBTB) in 410 patients with TBTB. Retrospective analysis was performed on the clinical features, bronchoscopy performance, bacteriological examination, imaging and treatment of 410 patients who were diagnosed with TBTB using bronchoscopy. Among the 410 patients, 10 patients underwent chest X-ray which revealed two cases of atelectasis, eight cases of patch or spot shadows, three cases of cavity, one case of nodule and one case with no abnormalities. The remaining 400 patients underwent computed tomography chest scans and/or airway reconstruction examinations. Among all the lesion types, the cavity type was found to be the most likely to cause bronchial stenosis or obstruction, with statistically significant differences when compared with the congestion, stenosis or scar lesion types (P<0.01). Moreover, for the cavity type, there were 194 sites of obstruction prior to therapy; however, only 23 sites of obstruction remained following therapy. Furthermore, there were 34 sites without stenosis prior to therapy and 205 sites without stenosis following therapy. The number of sites of obstruction was significantly decreased and the number of sites without stenosis was increased upon therapy. These findings suggest that the cavity type is the most sensitive type to therapy among the five types of TBTB lesion.

## Introduction

Tuberculous bronchitis is a submucosal tuberculosis mainly found in bronchial mucosa and submucosa (1,2). Tuberculous bronchitis has also been diagnosed in smooth muscle, cartilage and outer membranes; thus it is termed tracheobronchial tuberculosis (TBTB) (3). TBTB may be misdiagnosed as bronchitis, asthma, bronchiectasis or lung cancer (4,5). TBTB is present in 10%-40% of patients with active pulmonary tuberculosis (6) and the incidence of TBTB has been increasing in recent years (7). TBTB is more common in young adults than older ones, and females are more readily infected than males (8). The onset of TBTB may be concealed and its clinical manifestations are not specific, which makes the diagnosis of TBTB difficult. Clinically (9), TBTB patients may have systematic respiratory symptoms such as severe cough, sputum, wheezing, hemoptysis and paroxysmal dyspnea. In TBTB patients with stenosis in the trachea, left and right main bronchus, and middle segment of bronchus, the cough sounds like barking. In certain patients, these symptoms may also be accompanied by systematic symptoms (such as fever, night sweats, anorexia, fatigue, weight loss and irregular menstruation) and allergic manifestations (such as allergic arthritis and conjunctivitis).

In the present study, a retrospective analysis of 410 patients with TBTB was performed in order to investigate the characteristics and risk factors associated with TBTB. The patients selected for the present study were enrolled at Shandong Chest Hospital (Jinan, China) between January and December 2012.

## Materials and methods

### Patients

The present study was approved by the Ethics Committee of Shandong University (Jinan, China). All patients provided written informed consent. None of the patients were long-term users of hormones for the treatment of autoimmune diseases, including cancer, AIDS, diabetes*,* systemic lupus erythematosus and organ transplantation.

### Diagnosis

All patients underwent sputum acid-fast bacilli examination (three smears and one culture using the BACTEC method), electronic bronchoscopy and a chest X-ray or computed tomography (CT) scan. According to previously described criteria (3), the 410 patients met the following criteria: i) typical lesions of TBTB identified by bronchoscopy and ii) a positive sputum smear for acid-fast bacilli and/or a positive sputum culture for *Mycobacterium tuberculosis*; or bronchial brushing or bronchoalveolar lavage samples positive for acid-fast bacilli; or tuberculous pathological changes identified using bronchoscopic biopsy.

### Treatment

Among the 410 patients with TBTB, 382 patients were newly diagnosed. The remaining 28 cases were patients who required retreatment. The registered patients adopted the 3HRZE/6HRE program (H, isoniazid; R, rifampicin; Z, pyrazinamide; E, ethambutol). The 28 patients requiring retreatment received the 3HRZEL/6HREL program supplemented with 0.2 g isoniazid inhalation therapy. The 410 patients were treated with endobronchial injection, clamping, balloon dilatation, refrigeration and other treatments. The patients with severe airway obstruction were administered bronchoscopic therapy under general anesthesia 2–15 times. The patients were followed up for between 3 and 9 months using bronchoscopy.

### Bronchoscopy and TBTB typing

**Figure 1:**
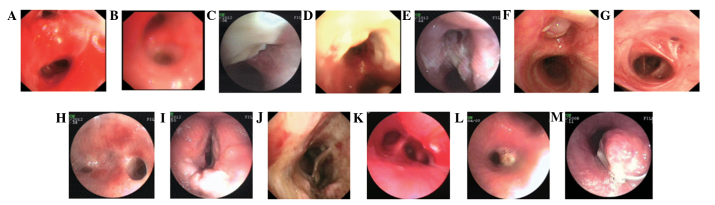
The six pathological types of tracheobronchial tuberculosis. The six types consist of the (A and B) inflammatory infiltration, (C and D) ulceration necrosis, (E and F) proliferative granulation type, (G and H) scar stenosis type, (I and J) wall softening type and (K–M) lymph fistula types.

The diagnosis of TBTB was performed using bronchoscopy and bacteriological or pathological analysis results. In China, TBTB is classified into six pathological types, including the inflammatory infiltration type (Fig. 1A and B), the ulceration necrosis type (Fig. 1C and D), the proliferative granulation type (Fig. 1E and F), the scar stenosis type (Fig. 1G and H), the wall softening type (Fig. 1I and J) and the lymph fistula type (Fig. 1K–M) (10).

### Imaging analysis

Among the 410 patients with TBTB, 10 patients received chest X-ray examination. The remaining 400 patients underwent spiral CT examination of the chest and/or airway reconstruction examinations.

### Statistical analysis

SPSS software, version 21.0 (SPSS, Inc., Chicago, IL, USA) was used for the statistical analyses. Student’s t-tests and χ^2^ test were used for analysis of the differences between groups. P<0.05 was considered to indicate a statistically significant difference.

## Results

### Characteristics of patients

The present study included 109 male and 301 female patients in the total 410 patients with TBTB (Table I). The difference (109/301) between the number of males and the females was found to be significant (t=-2.357; P<0.001). Among the patients, 10 underwent chest X-ray, revealing two cases of atelectasis, eight cases of patch or spot shadows, three cases of cavity, one case of nodule and one patient with no abnormalities. The remaining 400 patients underwent chest CT scan and/or airway reconstruction examination. The results revealed 112 cases of partial or whole left lung atelectasis, 248 cases of bronchial stenosis, 135 cases of bronchial lumen obstruction, 255 cases of patch shadows, 179 cases of spot shadows, 124 cases of cavity, 34 cases of nodules, 134 cases of hilar and mediastinal lymph node enlargment, 65 cases of hilar or mediastinal lymph node calcification and 10 patients with no abnormal alterations.

The bronchoscopy results indicated that among the 109 male patients, there was 1 congestion lesion, 16 granulation lesions, 58 cavity lesions, 29 stenosis lesions and 7 scar lesions among the five lesion types. Among the 301 female patients, there were 6 congestion lesions, 33 granulation lesions, 170 cavity lesions, 80 stenosis lesions and 24 scar lesions. Among all the lesion types, the cavity lesion type was found to be the most likely to cause bronchial stenosis or obstruction (Table II), with statistically significant differences observed compared with the congestion, stenosis and scar lesion types (P<0.01; Table II). These findings suggest that the cavity lesion type is the predominant risk factor.

### Comparison of the bronchoscopic therapies

The 410 patients with TBTB all received the aforementioned local treatments. There were statistically significant differences in the bronchial lumen for the granulation, cavity and congestion lesion types prior to and following therapy (P<0.01; Table III). However, there were no significant differences in bronchial lumen for the fibrous stenosis and scar lesions types prior to and following therapy, as shown in Table III. For the cavity type, there were 194 sites of obstruction prior to therapy and only 23 sites of obstruction following therapy (Table III). Furthermore, there were 34 sites without stenosis prior to therapy and 205 sites without stenosis following therapy. These results suggest that the cavity type is the most sensitive to therapy among the five lesion types. Moreover, the number of sites of obstruction was significantly decreased and the number of sites without stenosis was relatively increased following therapy.

## Discussion

In the present study, 400 patients underwent chest CT scan and/or airway reconstruction examinations, including 112 cases of partial or left whole lung atelectasis, 248 cases of bronchial stenosis and 135 cases of bronchial obstruction. X-ray examinations have no specificity for the diagnosis of TBTB (10,11). However, chest CT scans with airway reconstruction techniques are able to show stenosis and obstruction in the bronchial lumen and clearly demonstrate the extent and scope of the bronchial stenosis or obstruction, revealing the changes in the bronchial walls more clearly. In addition, using this method, it is possible to determine whether the hilar and mediastinal lymph nodes are enlarged or have calcification (6,13). Thus, CT scans with airway reconstruction techniques have clinical value for TBTB diagnosis.

In the present study, all patients underwent sputum smear microscopy three times in order to detect acid-fast bacilli and sputum culture once using the BACTEC method. There were 135 cases that were sputum smear positive and 177 cases that were sputum culture positive. All patients also underwent bronchoscopy and brushing for acid-fast bacilli sputum smear and mycobacterial culture one week following admission. There were 78 cases of patients who were acid-fast bacilli smear positive and 169 cases of patients who were brush mycobacterial culture positive among the 410 cases. There was no significant difference in the tuberculosis positive rate between the male and female patients.

TBTB is capable of invading bronchial tissue, submucosa, muscle, bronchial cartilage and outer membranes at many sites (3,14–16). Bronchiolar involvement has been found to be common in the main bronchus and upper lobe bronchus, and is more common in the left than in the right (13). In the present study, bronchoscopy revealed that among the patients with TBTB, 193 patients had lesions located in one site and 217 cases had lesions in multiple sites, predominantly in the left main bronchus (32.7%). A moderate number of lesions were observed in the left upper lobe bronchus and the lowest number was found in the right lower lobe bronchus.

Among the five types of lesion, bronchoscopy examination in the present study revealed 7 congestion lesions, 49 granulation lesions, 228 cavity lesions, 109 stenosis lesions and 31 scar lesions. Furthermore, cavity lesions were found to be significantly more common than the other types of lesions (P<0.01).

In conclusion, the incidence of TBTB is associated with gender and age. Young women usually have a higher incidence and more serious disease. Among all the types of lesions, the cavity lesion type has the highest incidence. In addition, the cavity type is more likely to cause bronchial stenosis or obstruction. Bronchoscopy is an effective means for the diagnosis and treatment of TBTB and early bronchoscopy intervention is likely to achieve better results.

## Figures and Tables

**Table I:** Comparison of lesion types in the 410 patients with tracheobronchial tuberculosis enrolled in the present study.

Gender	Total number of lesions, n (%)	Congestion, n (%)	Granulation, n (%)	Cavity, n (%)	Stenosis, n (%)	Scar, n (%)	χ^2^	P-value
Male (n=109)	111 (26.2)	1 (0.2)	16 (3.8)	58 (13.7)	29 (6.8)	7 (1.7)		
Female (n=301)	313 (73.8)	6 (1.4)	33 (7.8)	170 (40.1)	80 (18.9)	24 (5.7)		
Total (n=410)	424 (100)	7 (1.7)	49 (11.6)	228 (53.8)	109 (25.7)	31 (7.3)	1.858	0.762

χ^2^ value and the P-value were calculated through comparing the pathological lesion types between the male and the female groups.

**Table II:** Changes in the bronchial lumen caused by tracheobronchial tuberculosis.

	Changes in the bronchial lumen					
			
Lesion type	No stenosis, n (%)	Stenosis, n (%)	Obstruction, n (%)	Total, n (%)	χ^2^	P-value
Cavity	34 (5.1)	228 (34.4)	166 (25.1)	428 (64.7)		
Congestion	4 (0.6)	3 (0.5)	0 (0)	7 (1.1)	21.940	<0.001
Granulation	8 (1.2)	26 (3.9)	15 (2.3)	49 (7.4)	4.283	0.117
Stenosis	0 (0)	109 (16.5)	38 (5.7)	147 (22.2)	24.976	<0.001
Scar	11 (1.7)	18 (2.7)	2 (0.3)	31 (4.7)	30.743	<0.001
Total	57 (8.6)	384 (58.0)	221 (33.4)	662 (100)	85.583	<0.001

χ^2^ value, cavity type compared with other types; P-value, cavity types compared with other types.

**Table III:** Changes in the bronchial lumen prior to and following therapy.

Lesion type	-Lesion number (total 424)	Prior to therapy		Following therapy		χ^2^	P-value
	
		No stenosis, n (%)	Stenosis and/or obstruction, n (%)	No stenosis, n (%)	Stenosis and/or obstruction, n (%)		
Cavity	228	34 (8.0)	194 (45.8)	205 (48.3)	23 (5.4)		
Congestion	7	4 (0.9)	3 (0.7)	7 (1.7)	0 (0)	470.000	<0.001
Granulation	49	8 (1.9)	41 (9.7)	44 (10.4)	5 (1.2)	554.000	0.009
Stenosis	109	0 (0)	109 (25.7)	58 (13.7)	51 (12.0)	674.000	<0.001
Scar	31	11 (2.6)	20 (4.7)	11 (2.6)	20 (4.7)	518.000	<0.001

χ^2^, cavity type compared with other types. P, cavity type compared with other types.
